# Tight junction-related proteins in the upper reproductive tract luminal and glandular epithelial cells are regionally regulated across the estrous cycle

**DOI:** 10.3389/fendo.2026.1865472

**Published:** 2026-08-17

**Authors:** Donaldson A. Magloire, Haoming Liu, Heather L. Wilson

**Affiliations:** 1 Vaccine and Infectious Disease Organization, University of Saskatchewan, Saskatoon, SK, Canada; 2 Department of Veterinary Microbiology, Western College of Veterinary Medicine, University of Saskatchewan, Saskatoon, SK, Canada; 3 Vaccinology and Immunotherapeutics Program, School of Public Health, University of Saskatchewan, Saskatoon, SK, Canada

**Keywords:** Claudin-4, Claudin-3, Zonula occludens-1, immunofluorescence, tight junctions, reproductive immunology, upper reproductive tract, estrous cycle

## Abstract

**Introduction:**

The immune system in the upper reproductive tract (URT) protects against sexually transmitted pathogens, while at the same time providing immune tolerance responses against allogenic sperm and the developing fetus. Estrus is the time point at which the cervix is most receptive to breeding and when intrauterine immunization is typically administered.

**Methods:**

The aim of the present study was to use confocal immunofluorescence to assess the intensity and cellular localization of Tight Junction (TJ) proteins Claudin-3 and Claudin-4 and TJ scaffold protein Zonula occludens-1 (ZO-1) in the luminal epithelium (LE) and glandular epithelium (GE) in the distal and medial uterine horn and the cervix, during diestrus and during estrus.

**Results:**

Claudin-4 expression was significantly reduced during estrus relative to diestrus in the distal uterine horn, with the exception of the cervix, where expression was unchanged between stages. Claudin-3 exhibited a broader pattern of estrous stage-associated reduction, with significantly lower expression during estrus than diestrus in the glandular epithelium of the distal uterine horn, in both epithelial compartments of the medial uterine horn, and in the cervical luminal epithelium. ZO-1 localization and expression were largely unaffected by estrous stage across all regions examined.

**Conclusion:**

Together, these data reveal estrous stage-dependent and region-specific regulation of Claudin-4 and Claudin-3 consistent with selective remodeling without scaffold disruption in the porcine reproductive tract. To our knowledge, this is the first multi-regional analysis of tight junction protein expression across the distal uterine horn, medial uterine horn, and cervix of the porcine reproductive tract during the estrous cycle. These changes indicate modulation of tight junction composition that may correspond to altered epithelial barrier properties at estrus.

## 1 Introduction

The upper reproductive tract (URT) must balance two competing demands: defense against ascending pathogens and tolerance of allogenic sperm and the developing fetus. The epithelial barrier is central to this balance, and tight junctions (TJs) are the principal structures that maintain its integrity, sealing the intercellular space between adjacent epithelial cells and restricting uncontrolled diffusion of solutes and macromolecules (1). Although limited lymphoid aggregates and cyclical immune cell recruitment have been described within the porcine URT (2–8), the mechanisms by which luminal antigens interact with underlying immune populations remain incompletely understood. Despite the importance of the epithelial barrier in mediating this balance, the mechanisms regulating its properties across the estrous cycle are similarly not well defined.

The ovarian steroid hormones estradiol and progesterone drive cyclical changes in uterine epithelial structure and function across the estrous cycle (9). Beyond their reproductive roles, these hormones are increasingly recognized as regulators of epithelial barrier properties in reproductive tract tissues (10–12). Hormonal regulation of barrier function has been linked to changes in tight junction organization and claudin expression in multiple epithelial tissues (13–17). Recent studies have further demonstrated that epithelial barriers are highly dynamic structures whose properties are regulated through hormone-sensitive signaling pathways that influence tight junction composition and cellular polarity rather than through complete disruption of junctional architecture (18–20). Thus, hormonal signaling represents a plausible mechanism for region- and stage-specific modulation of epithelial barrier properties within the porcine URT. Hormonally driven changes in epithelial barrier properties are thought to occur through remodeling of the claudin composition of the TJ complex itself, rather than through wholesale disassembly of the junction, pointing to hormone-driven barrier plasticity as a theme in reproductive tract epithelia (21).

Claudins, a multigene family of ~23 kDa tetraspan transmembrane proteins, are the principal determinants of TJ selectivity. Multiple claudin isoforms are typically co-expressed within a single TJ complex, and the specific combination of claudin proteins dictates the permeability properties of the paracellular pathway (22). Once polymerized into TJ strands, claudins are stabilized through interactions with zonula occludens-1 (ZO-1) scaffold proteins. ZO-1 serves as a key adaptor protein that links transmembrane tight junction components to the actin cytoskeleton and organizes structurally diverse yet functionally interconnected proteins at the junctional complex (23). Emerging evidence suggests that selective modulation of claudin composition represents a major mechanism by which epithelial tissues fine-tune barrier properties in response to physiological and environmental cues while maintaining overall tight junction organization (24–26). In addition to their barrier function, TJs maintain epithelial polarity by acting as a molecular fence that prevents the free diffusion of proteins and lipids between apical and basolateral membrane domains (27).

However, whether TJ regulation in the porcine URT varies across distinct anatomical compartments of the reproductive tract, and whether this spatial heterogeneity tracks with estrous stage, remains unknown. A comprehensive comparison of Claudin-3, Claudin-4, and ZO-1 expression across the distal uterine horn, medial uterine horn, and cervix has not been reported, despite the pig’s relevance as a model for human reproductive immunology and its direct application to intrauterine vaccination practice.

The present study aimed to characterize the spatial expression patterns of Claudin-3, Claudin-4, and ZO-1 throughout the porcine URT during estrus and diestrus. We hypothesized that expression of the barrier-forming claudins, but not the scaffold protein ZO-1, would differ between estrus and diestrus in a region-specific manner across the distal uterine horn, medial uterine horn, and cervix, reflecting selective rather than global remodeling of the tight junction complex.

## 2 Materials and methods

### 2.1 Animal use and ethics

All experimental procedures were approved by the University of Saskatchewan Animal Research Ethics Board and performed according to the guidelines of the Canadian Council on Animal Care (CCAC). Uteri from pigs in estrus (n = 4) were obtained from Prairie Swine Centre Inc. (PSC), and uteri from pigs in diestrus (n = 4) were collected from a local abattoir. For PSC animals, estrus was determined by PSC staff using standard behavioral and visual signs, including the standing reflex in the presence of a boar and vulval swelling. For abattoir-sourced animals, diestrus was confirmed at necropsy by the presence of functional corpora lutea on the ovaries. We therefore refer to “estrous stage-associated” rather than “hormone-dependent” or “hormonal” regulation throughout this manuscript, as circulating hormone concentrations were not directly measured. Sample size is consistent with prior studies characterizing protein expression in the porcine uterine epithelium (28). Tissue sections were excised from three anatomical regions of the uterine horn: (i) approximately 20 cm from the distal end of the horn (distal uterine horn), (ii) the midpoint of the uterine horn (medial uterine horn), and (iii) the cervical region adjacent to the uterine body (Cervix). Tissues were fixed in 10% neutral-buffered formalin and subsequently paraffin-embedded by Prairie Diagnostic Services Inc.

### 2.2 Immunofluorescent staining

Paraffin-embedded uterine tissues were sectioned at 5 µm and mounted onto Superfrost™ Plus microscope slides (Fisher Scientific). Sections were deparaffinized in xylene (4 × 5 min) and rehydrated through a graded ethanol series followed by distilled water. Heat-induced epitope retrieval (HIER) was performed in 10 mM Tris, 1 mM EDTA (pH 9.0) for 15 min. Slides were allowed to cool to room temperature and washed in phosphate-buffered saline containing 0.05% Tween-20 (PBST). Sections were incubated overnight at 4 °C with the following primary antibodies diluted 1:100 in blocking buffer: Claudin-4 (Thermo Fisher Scientific, 36-4800),

Claudin-3 (Thermo Fisher Scientific, 34-1700), ZO-1 (Thermo Fisher Scientific, 33-9100). After washing, sections were incubated for 1 h at room temperature with the appropriate secondary antibodies diluted 1:1000: Goat anti-rabbit Alexa Fluor™ 488 (Thermo Fisher Scientific, A-11008), Goat anti-mouse Alexa Fluor™ 568 (Thermo Fisher Scientific, A-21124). Slides were mounted using ProLong™ Gold Antifade Mountant with DAPI (Thermo Fisher Scientific, P36935) and coverslipped with #1.5 glass coverslips (Fisher Scientific). Tissues known to express the target proteins were included as positive controls and processed alongside experimental samples. Negative controls without primary antibody and isotype-matched IgG controls were also included. These control sections showed minimal background staining. Imaging settings were kept consistent across all samples to allow reliable quantitative comparison.

### 2.3 Hematoxylin and eosin staining

Paraffin-embedded uterine tissues were sectioned at 5 µm, deparaffinized in xylene, and rehydrated through a graded ethanol series. Sections were stained with hematoxylin and eosin using standard histological procedures, dehydrated through graded ethanol, cleared in xylene, and coverslipped. Brightfield images were acquired using identical microscope settings for all samples to document tissue morphology and histological architecture prior to immunofluorescence analysis.

### 2.4 Immunohistochemistry

Chromogenic immunohistochemistry (IHC) was performed as an independent qualitative method to validate the tissue localization patterns observed by confocal immunofluorescence. The same primary antibodies used for immunofluorescence were employed for IHC to confirm the specificity and anatomical localization of Claudin-3, Claudin-4, and ZO-1.

Following deparaffinization and rehydration, tissue sections were incubated in 3% hydrogen peroxide (H_2_O_2_) in methanol for 10 min to quench endogenous peroxidase activity. Heat-induced epitope retrieval (HIER) was performed in Tris–EDTA buffer (pH 9.0) for 15 min. Sections were incubated overnight at 4 °C with the following primary antibodies diluted 1:100 in blocking buffer: Claudin-4 (Thermo Fisher Scientific, 36-4800), Claudin-3 (Thermo Fisher Scientific, 34-1700), and ZO-1 (Thermo Fisher Scientific, 33-9100). After washing, sections were incubated with HRP-conjugated secondary antibodies (Dako EnVision™ anti-rabbit HRP, K4003; anti-mouse HRP, K4001) according to the manufacturer’s instructions. Signal was developed using 3,3′-diaminobenzidine (DAB) chromogen for 10 min. Slides were toned in 2% copper sulfate, counterstained with hematoxylin, dehydrated through graded ethanol, cleared in xylene, and coverslipped.

Because IHC was performed to validate protein localization rather than quantify protein abundance, no quantitative or semi-quantitative analysis of the chromogenic staining was performed. Quantitative comparisons of protein expression were based exclusively on mean fluorescence intensity measurements obtained by confocal immunofluorescence.

### 2.5 Confocal microscopy acquisition

High-resolution confocal microscopy was performed to quantify fluorescence intensity and assess epithelial localization patterns across anatomical sites and estrus stages. Fluorescent images were acquired using a Leica TCS SP8 laser scanning confocal microscope equipped with a 63×/1.4 NA oil immersion objective. Images were collected at 2048 × 2048 pixel resolution (approximate pixel size ~90 nm), with a dwell time of 1.2 µs, line averaging of 2, and sequential line scanning to minimize spectral overlap. Quantification was performed on single optical sections (no maximum intensity projections were used). Images were processed and analyzed using QuPath software.

### 2.6 Fluorescence intensity measurements

For each animal, three regions of interest (ROIs) were placed along either the LE or GE, based on continuous, well-oriented epithelium with clear apical staining; regions with tissue folds, edge artifacts, or visible damage were excluded, as were stromal regions. ROI size and placement were kept consistent across all samples to ensure comparability. Mean fluorescence intensity (MFI) values for Claudin-3 and Claudin-4 were measured within each ROI, averaged per animal, and used for statistical analysis. Slides were coded prior to imaging and analysis, and ROI selection, image acquisition, and MFI quantification were performed blind to estrous stage.

### 2.7 Statistical analysis

For all experiments, n = 4 represents the number of individual sows per group. Statistical analyses were performed using GraphPad Prism. Comparisons between estrus and diestrus groups were performed using the Mann–Whitney U test. Comparisons across the three anatomical regions were analyzed using the Kruskal–Wallis test followed by Dunn’s multiple comparisons *post hoc* test. Non-parametric tests were selected given the small sample size (n = 4 per group), for which normality cannot be reliably assessed and parametric assumptions cannot be verified. Data are presented as mean ± SEM. Statistical significance was defined as p < 0.05.

## 3 Results

### 3.1 Histological assessment

**Figure 1:**
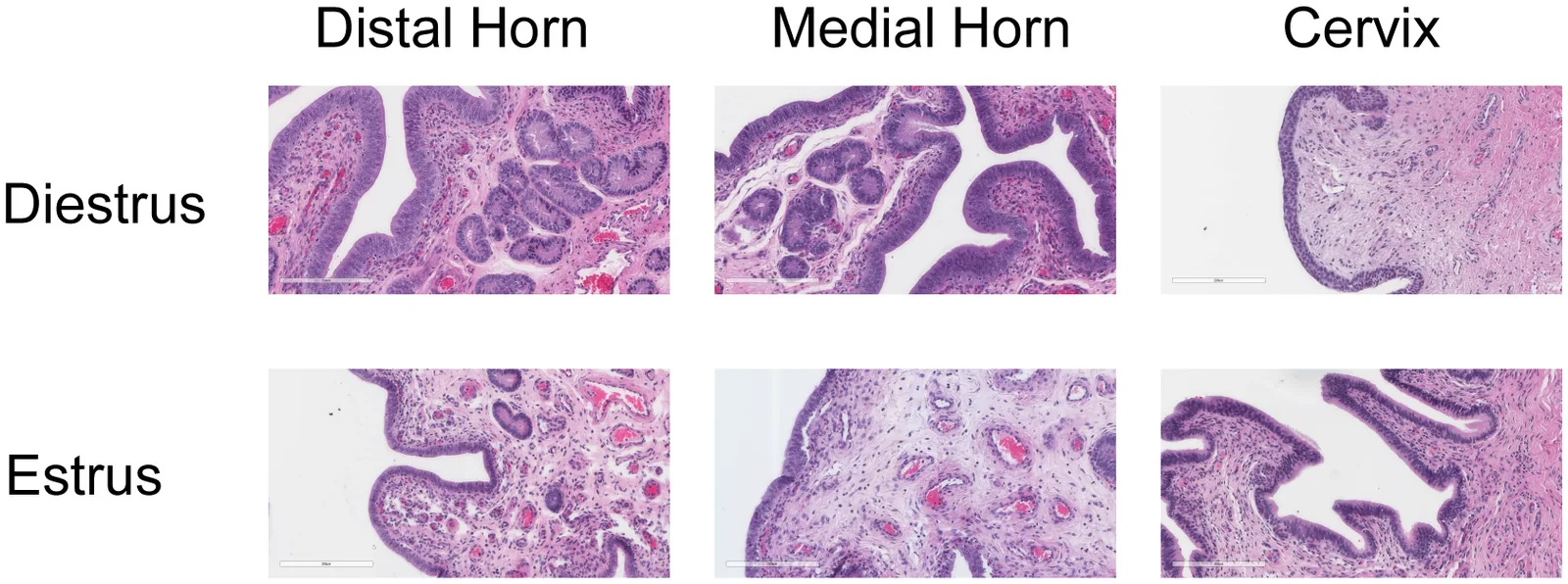
Hematoxylin and eosin (H&E) morphology of the porcine distal uterine horn, medial uterine horn, and cervix. Representative H&E-stained sections illustrating the histological architecture of the distal uterine horn, medial uterine horn, and cervix. The luminal epithelium (LE), glandular epithelium (GE), and stromal compartments are visible, demonstrating preserved tissue morphology and structural integrity prior to immunofluorescence analysis. Images were acquired using brightfield microscopy under identical acquisition settings. Scale bar = 200 µm.

In this study, we investigated the TJ protein expression using pigs from estrus and diestrus. We isolated regions of the distal uterine horn, medial uterine horn and cervix. Hematoxylin and eosin (H&E) staining was performed to confirm preservation of tissue architecture across all uterine regions and estrous stages (**Figure 1**). The LE, GE and stromal compartments appeared morphologically intact in samples obtained at both diestrus and estrus, supporting suitability for downstream immunofluorescence analysis.

### 3.2 Claudin-4 exhibits region-specific regulation across the estrous cycle

**Figure 2:**
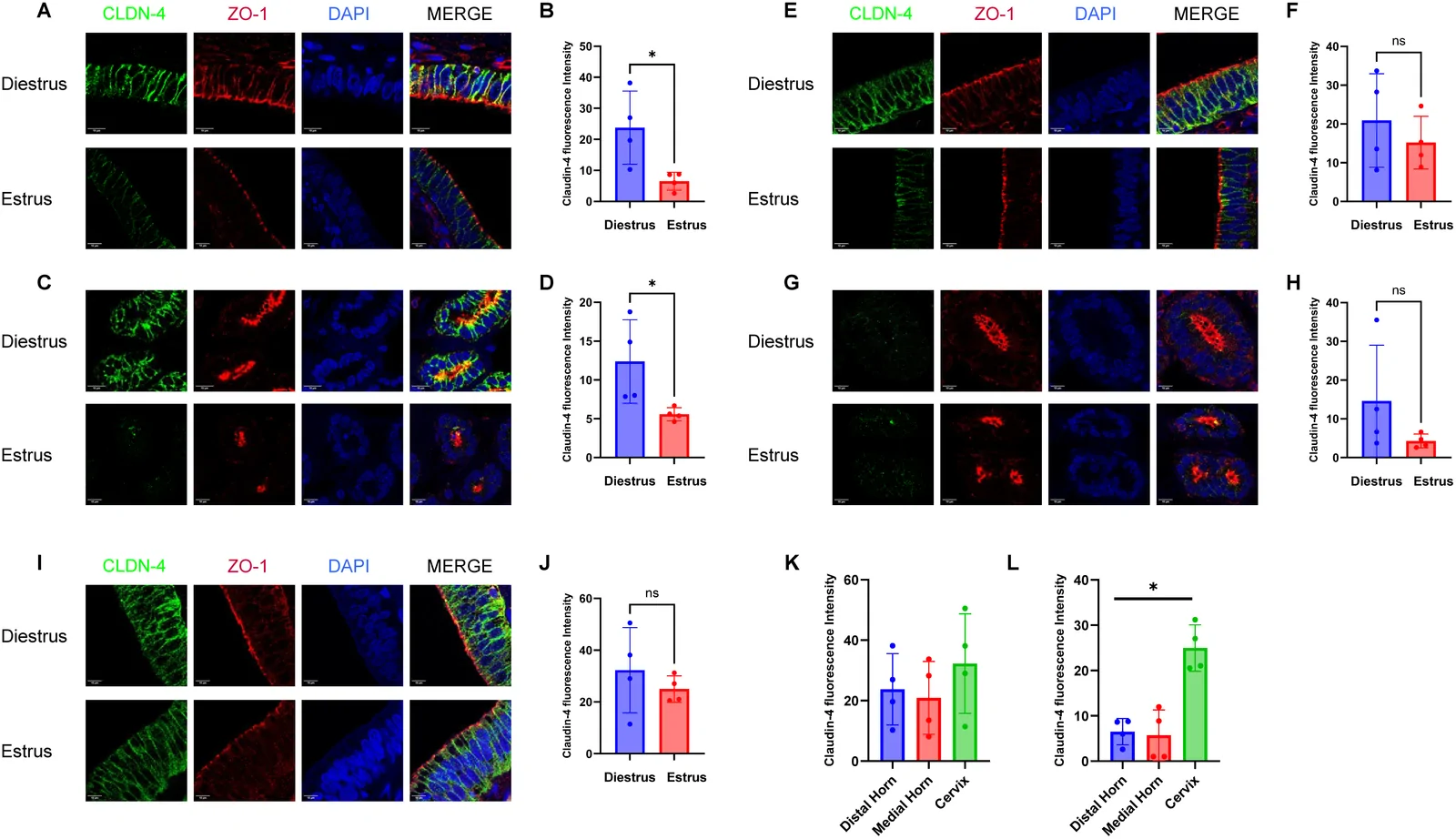
Claudin-4 expression and regional comparison in the porcine upper reproductive tract during diestrus and estrus. **(A–D)** Distal uterine horn. Representative confocal images of Claudin-4 localization in the luminal epithelium [LE; **(A)**] and glandular epithelium [GE; **(C)**], together with corresponding MFI quantification **(B, D)**. Claudin-4 MFI was significantly higher during diestrus than estrus in both LE and GE. **(E–H)** Medial uterine horn. Representative confocal images of Claudin-4 localization in the LE **(E)** and GE **(G)**, together with corresponding MFI quantification **(F, H)**. Claudin-4 MFI did not differ significantly between estrous stages in either epithelial compartment. **(I–J)** Cervix. Representative confocal image **(I)** and corresponding MFI quantification **(J)** of Claudin-4 in the luminal epithelium. Claudin-4 expression did not differ significantly between estrous stages. Glandular epithelium was not assessed in the cervix, as the porcine cervical mucosa lacks true glandular structures. **(K–L)** Regional comparison of Claudin-4 MFI across the distal uterine horn, medial uterine horn, and cervix during diestrus **(K)** and estrus **(L)**. Claudin-4 (green), ZO-1 (red), and nuclei (DAPI, blue). Scale bars = 10 µm. Images were acquired using a Leica TCS SP8 confocal microscope under identical acquisition settings across all samples. Data are presented as mean ± SEM (n = 4 sows/group). Statistical significance between estrus and diestrus was determined using the Mann–Whitney U test; regional comparisons were analyzed using the Kruskal–Wallis test with Dunn’s *post hoc* correction (*p < 0.05).

Claudin-4 expression and localization were assessed in the LE and GE of the distal uterine horn, medial uterine horn, and cervix. Claudin-4 expression in the LE of the distal uterine horn was significantly higher during diestrus compared to estrus (**Figures 2A, B**). A similar pattern was observed in the GE, where Claudin-4 intensity was significantly elevated in diestrus relative to estrus (**Figures 2C, D**). In diestrus tissues, Claudin-4 exhibited continuous apical junctional staining in the LE, whereas estrus samples showed reduced fluorescence intensity (**Figure 2A**). A similar reduction in fluorescence intensity was observed in the GE during estrus (**Figure 2C**).

In the medial uterine horn, Claudin-4 intensity was higher in diestrus than estrus in both LE and GE (**Figures 2E, G**); however, these differences did not reach statistical significance (**Figures 2F, H**).

In the cervix, Claudin-4 expression did not differ between estrous stages (**Figure 2I**) such that the stained tissues exhibited bright, continuous apical staining in the LE in both estrus and diestrus (**Figure 2J**).

Regionally, Claudin-4 expression during diestrus was numerically highest in the cervix relative to the medial and distal uterine horn, although this regional difference did not reach statistical significance (**Figure 2K**). In contrast, during estrus, Claudin-4 expression differed significantly across the three regions examined (**Figure 2L**). Claudin-4 localization patterns were confirmed by chromogenic IHC (**Supplementary Figure 1A**).

### 3.3 Claudin-3 expression level is reduced during estrus across multiple uterine regions

**Figure 3:**
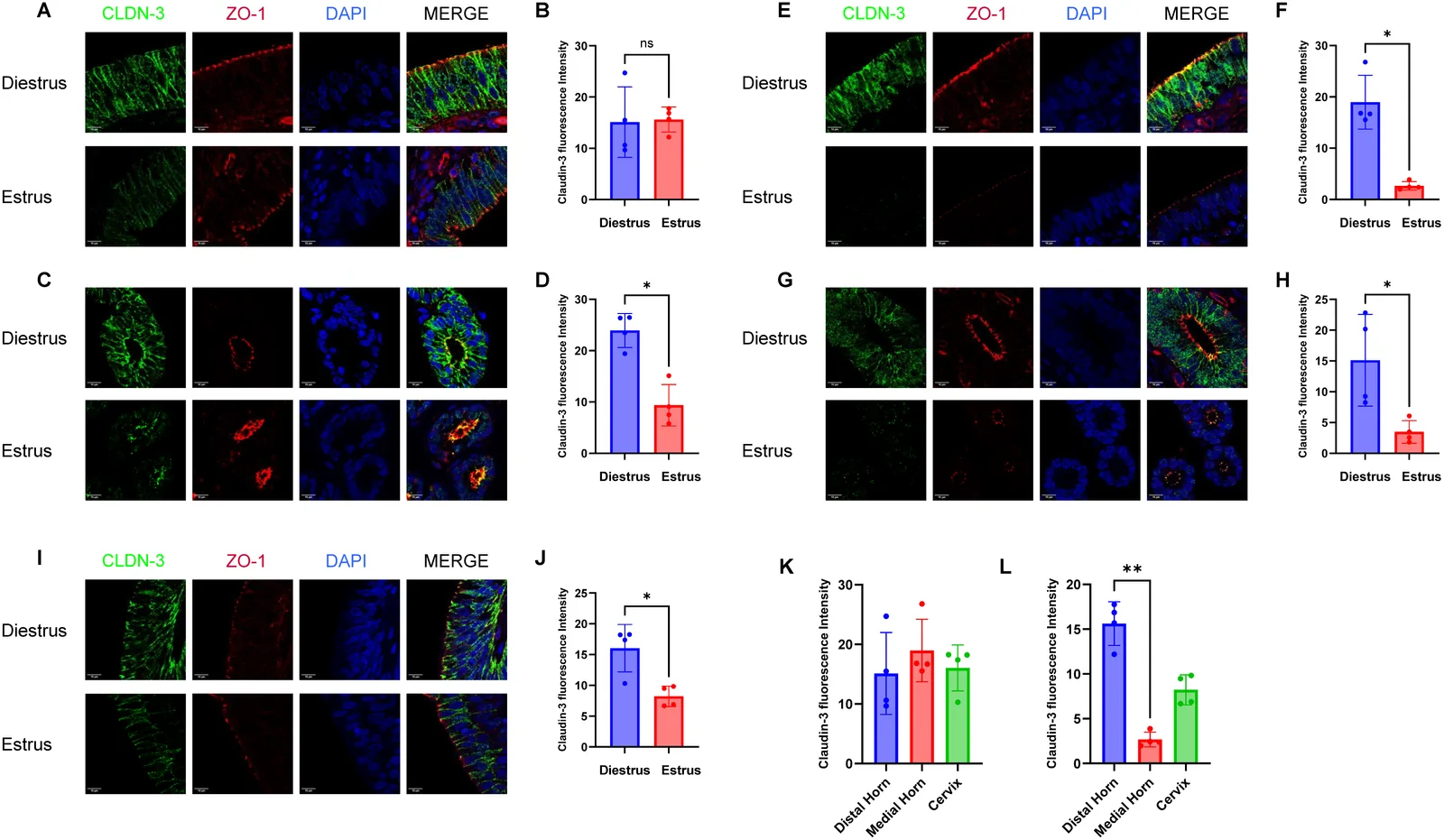
Claudin-3 expression and regional comparison in the porcine upper reproductive tract during diestrus and estrus. **(A–D)** Distal uterine horn. Representative confocal images of Claudin-3 localization in the luminal epithelium [LE; **(A)**] and glandular epithelium [GE; **(C)**], together with corresponding MFI quantification **(B, D)**. Claudin-3 MFI did not differ significantly between estrous stages in the LE, but was significantly higher during diestrus than estrus in the GE. **(E–H)** Medial uterine horn. Representative confocal images of Claudin-3 localization in the LE **(E)** and GE **(G)**, together with corresponding MFI quantification **(F, H)**. Claudin-3 MFI was significantly higher during diestrus than estrus in both epithelial compartments. **(I–J)** Cervix. Representative confocal image **(I)** and corresponding MFI quantification **(J)** of Claudin-3 in the luminal epithelium. Claudin-3 MFI was significantly higher during diestrus than estrus. Glandular epithelium was not assessed in the cervix, as the porcine cervical mucosa lacks true glandular structures. **(K–L)** Regional comparison of Claudin-3 MFI across the distal uterine horn, medial uterine horn, and cervix during diestrus **(K)** and estrus **(L)**. Claudin-3 (green), ZO-1 (red), and nuclei (DAPI, blue). Scale bars = 10 µm. Images were acquired using a Leica TCS SP8 confocal microscope under identical acquisition settings across all samples. Data are presented as mean ± SEM (n = 4 sows/group). Statistical significance between estrus and diestrus was determined using the Mann–Whitney U test; regional comparisons were analyzed using the Kruskal–Wallis test with Dunn’s *post hoc* correction (*p < 0.05, **p < 0.01).

Claudin-3 localization was assessed in the LE and GE of the distal uterine horn, medial uterine horn, and cervix to evaluate estrous stage-associated regulation of this TJ protein. In the distal uterine horn, Claudin-3 expression in the LE did not significantly differ between estrus and diestrus (**Figure 3A**). Although variation in LE fluorescence intensity was observed (**Figure 3B**), these differences did not reach statistical significance. In contrast, Claudin-3 intensity in the GE was significantly reduced during estrus compared to diestrus (**Figures 3C, D**). In the medial uterine horn, Claudin-3 expression was significantly downregulated during estrus compared to diestrus in both epithelial compartments of LE and GE (**Figures 3E–H**). In the cervix, Claudin-3 expression in the LE was significantly elevated during diestrus relative to estrus (**Figures 3I, J**).

Across all anatomical regions, Claudin-3 remained predominantly localized to the apical junctional region of epithelial cells regardless of estrous stage; however, staining intensity was reduced during estrus in most regions examined (**Figures 3K, L**). Chromogenic IHC demonstrated comparable epithelial localization patterns (**Supplementary Figure 1B**).

### 3.4 ZO-1 expression is stable across the estrous cycle and uterine regions

**Figure 4:**
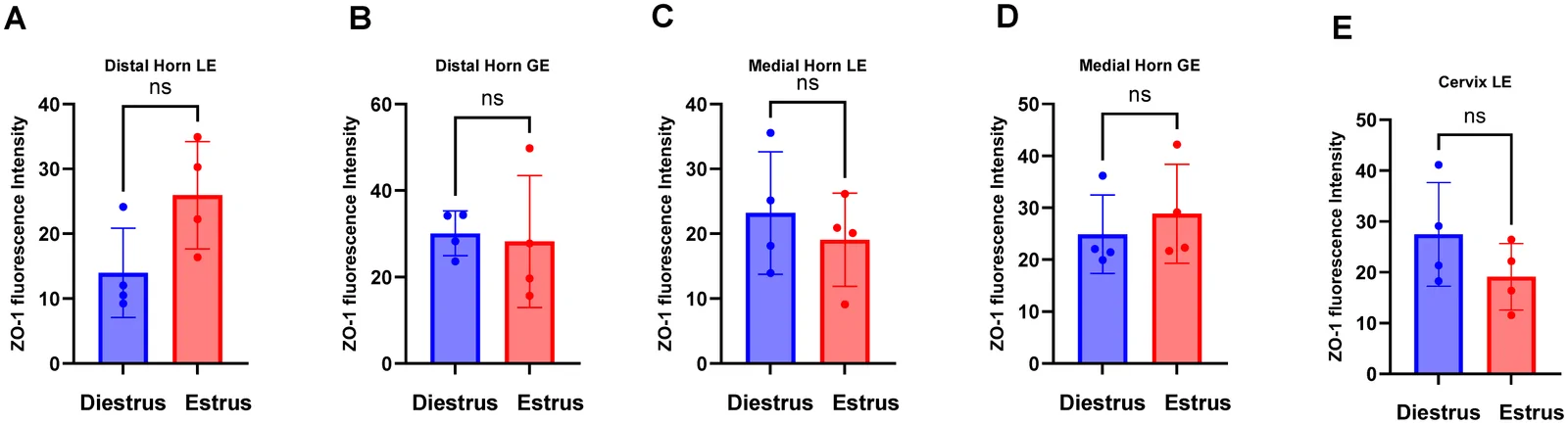
ZO-1 expression across uterine regions during diestrus and estrus. **(A, B)** Distal uterine horn. MFI quantification of ZO-1 in the luminal epithelium **(A)** and glandular epithelium **(B)**. ZO-1 MFI did not differ significantly between estrous stages in either epithelial compartment. **(C, D)** Medial uterine horn. MFI quantification of ZO-1 in the luminal epithelium **(C)** and glandular epithelium **(D)**. ZO-1 MFI did not differ significantly between estrous stages in either epithelial compartment. **(E)** Cervix. MFI quantification of ZO-1 in the luminal epithelium. ZO-1 MFI did not differ significantly between estrous stages. Glandular epithelium was not assessed in the cervix, as the porcine cervical mucosa lacks true glandular structures. Across all regions and estrous stages, ZO-1 remained localized to the apical junctional region of epithelial cells, consistent with the representative confocal images shown in **Figures 2** and **3**. Data are presented as mean ± SEM (n = 4 sows/group). Statistical significance between estrus and diestrus was determined using the Mann–Whitney U test (p < 0.05).

ZO-1 localization was evaluated to determine whether the estrous stage affected tight junction scaffold integrity. In the LE and GE of both the distal and medial uterine horn, ZO-1 fluorescence intensity did not significantly differ between estrus and diestrus (**Figures 4A–D**). Similarly, no significant differences in ZO-1 fluorescence intensity were detected in the cervical LE (**Figure 4E**). Across all regions and stages, ZO-1 remained localized to the apical junctional region of epithelial cells, and tight junctional staining appeared continuous and comparable between estrus and diestrus samples. Similar apical junctional localization was observed by DAB-based IHC (**Supplementary Figure 1C**).

## 4 Discussion

In this research, we demonstrated that TJ composition within the porcine upper reproductive tract is selectively and regionally modulated across the estrous stages. Importantly, changes were confined to barrier-forming Claudin-3 and Claudin-4, while the scaffold protein ZO-1 remained stable across anatomical regions and estrous stages. These findings suggest that the estrous cycle may drive controlled remodeling of TJ components rather than global disruption of TJ architecture. These two protein classes appear to occupy distinct functional roles within the TJ complex: ZO-1 provides scaffold stability by anchoring the junction to the cytoskeleton, while Claudin-3 and Claudin-4 act as barrier-tuning subunits whose relative abundance is more dynamically regulated. Our data are consistent with a model in which estrous stage influences the barrier component of the junction while leaving its structural scaffold intact.

Claudin-4 exhibited region-specific regulation, with significant downregulation during estrus in both the luminal and glandular epithelium of the distal uterine horn. In contrast, Claudin-3 demonstrated a broader pattern of estrous stage-associated downregulation, with reduced expression during estrus in both epithelial compartments of the distal and medial uterine horn and in the cervical luminal epithelium. The differential regulation of these claudins suggests isoform-specific contributions to epithelial barrier tuning along the reproductive tract (25).

The preservation of ZO-1 expression and localization across all regions supports maintenance of junctional scaffold integrity. Because ZO-1 anchors transmembrane tight junction proteins to the actin cytoskeleton (29), its stability suggests that estrous stage alters TJ composition without compromising epithelial cohesion. Together, these data support a model of selective claudin remodeling rather than tight junction disassembly.

The mechanistic basis for estrous stage-associated claudin remodeling observed here was not directly tested in this study, but several pathways are plausible candidates.

Estrogen receptor signaling has been shown to regulate claudin transcription and TJ strand composition in other epithelial tissues, while progesterone signaling is independently implicated in modulating endometrial claudin expression during the receptive phase of the reproductive cycle (19, 30–33). In addition, local cytokine signaling can drive dynamic claudin reorganization within an intact TJ complex without requiring junctional disassembly (20). Although the present study did not measure circulating hormone concentrations, local receptor expression, or cytokine activity, these pathways offer testable mechanistic hypotheses for the estrous stage-associated claudin changes reported here and represent a logical direction for future work in this model.

These findings are consistent with apical junctional localization of Claudin-4 previously reported in porcine intestinal epithelium (34) and extend these observations to the uterine horn across the estrous cycle. In bovine endometrium, differential expression of claudin family members across the estrous cycle has also been reported (35), indicating that hormonally regulated barrier plasticity may be conserved among livestock species. Rodent studies similarly show stable ZO-1 localization across estrous stages with stage-dependent variation in Claudin-3 abundance at TJs (36). In diestrus, Claudin-3 signaling appeared to line the uppermost portion of the LE whereas in estrus, most Claudin-3 signaling disappeared with only punctuated staining in the luminal surface of the plasma membrane (36). In human endometrial tissue, there was a weak or absent signal for Claudin-3 and Claudin-4 staining in the proliferative and secretory phase, and they were predominantly localized to the GE cell membrane (37). In contrast, we found that the porcine upper reproductive tract maintains elevated claudin levels during diestrus, suggesting that pigs regulate their epithelial barrier in a species-specific manner, potentially reflecting the structural requirements of their non-invasive placentation.

Notably, the cervix behaved as a distinct regulatory domain rather than following the pattern observed in the uterine horn: Claudin-4 expression in the cervix did not differ between estrus and diestrus, in contrast to its significant downregulation during estrus in the distal uterine horn. This suggests that the cervix may be comparatively resistant to estrous stage-associated claudin remodeling, consistent with its distinct anatomical and functional role as the entry point to the uterine cavity, where barrier integrity may be preferentially maintained across the cycle.

The relative stability of claudin-4 expression in the cervix across the estrous cycle further supports its role as a consistently protective barrier, likely minimizing ascending infection independent of cycle stage.

Taken together, our data suggest that tight junction composition changes according to estrous stage in a pattern broadly conserved across species. In our study, the reduced expression of Claudin-3 and Claudin-4 in LE during estrus indicates structural modulation of the tight junction complex in the pig uterus. Stable expression of ZO-1 indicates that overall TJ structure remains intact, suggesting that barrier adjustments may occur without compromising epithelial integrity. This change in Claudin-3 and Claudin-4 expression indicates that the tight junction complex is compositionally distinct between the estrus and diestrus uterus.

Future studies should directly assess epithelial barrier function and determine whether estrous stage-associated changes in tight junction composition influence the movement of luminal molecules across the uterine epithelium. Overall, our findings demonstrate that claudin expression in the porcine reproductive tract is both region-specific and estrous stage-associated, highlighting the dynamic nature of epithelial barrier regulation across the estrous cycle. Although we did not directly measure epithelial permeability in this study, future work using functional approaches such as transepithelial resistance measurements or tracer diffusion assays such as fluorescent dextran will be necessary to determine whether these changes translate into measurable alterations in paracellular transport across the upper porcine reproductive tract.

Our previous work demonstrated that intrauterine vaccination administered at breeding induces both local and systemic antigen-specific immune responses (8, 38, 39). Because vaccination occurs during estrus (when the cervix is more receptive to breeding), epithelial barrier properties at this stage are of primary functional relevance. The reduction in Claudin-3 and Claudin-4 expression during estrus, particularly within the distal uterine horn, indicates a structurally distinct epithelial barrier composition at this stage. Whether these compositional differences influence antigen movement across the uterine epithelium remains unknown and will require direct functional investigation.

Effective immune priming requires antigen uptake and presentation by antigen-presenting cells (APCs) within lymphoid structures (40). In the porcine uterus, macrophages and dendritic cells have been reported to be more broadly distributed throughout the endometrium during estrus, while localized deeper within the lamina propria at other stages (41, 42). This redistribution occurs concurrently with the estrous stage-associated changes in Claudin expression reported here. Whether these phenomena are functionally linked, and whether they influence antigen movement within the uterus, remains unknown and requires direct experimental investigation.

Unlike the human uterus, where epithelial cells can express MHC II molecules (13), porcine uterine epithelial cells do not express SLA-DRA, the porcine equivalent of MHC II (8), suggesting that antigen presentation is restricted to professional APCs. This lack of epithelial MHC II expression suggests that interactions between luminal antigens and stromal APCs likely depend on mechanisms other than direct antigen presentation by epithelial cells. However, the pathways by which luminal antigens reach underlying immune populations were not investigated in the present study and remain to be determined.

The targeted downregulation of Claudin-3 and Claudin-4 we observed during estrus coincides temporally with the timing of intrauterine vaccine administration; whether this structural change translates into a functionally altered window for antigen passage remains to be determined. However, stimulation of porcine uterine epithelial cells with Poly I:C increases expression of IFNβ, TNFα, CCL2, and CCL4 (8), indicating that these cells may contribute to immune signaling in response to vaccine adjuvants. Future studies incorporating spatial analysis of MHC II_+_ APC localization alongside epithelial barrier measurements in response to cytokines or hormones will further clarify the relationship between Claudin remodeling, epithelial permeability, and vaccine-induced immune activation.

This study has several limitations that should be considered when interpreting these findings. First, the sample size (n = 4 sows per group) reflects the limited availability of uteri at defined estrous stages and was not informed by a formal power analysis; several regional comparisons that did not reach statistical significance may be underpowered to detect true differences, and this should be confirmed in studies with larger cohorts. Second, estrus and diestrus tissues were obtained from different source populations (a research herd and an abattoir, respectively), introducing the possibility that differences in post-mortem handling time or tissue processing, rather than estrous stage alone, contributed to the observed differences in TJ protein expression; we cannot fully exclude this confound with the present design. Third, fluorescence quantification was performed on single optical sections rather than full z-stack volumes, which may not fully capture the three-dimensional architecture of the tight junction complex. Finally, and most importantly, this study characterized TJ protein expression and localization but did not directly assess epithelial barrier function; claims regarding paracellular permeability or antigen passage remain hypotheses to be tested with functional assays such as transepithelial electrical resistance or tracer flux measurements.

## 5 Conclusion

Across the estrous cycle, Claudin expression changes in a region-specific manner within the porcine upper reproductive tract, while the overall TJ structure remains intact. Claudin-4 expression was significantly reduced during estrus in the LE and GE of the distal uterine horn, but not in the medial uterine horn or the cervix. Claudin-3 showed a broader pattern of estrous stage-associated reduction across multiple uterine regions. In contrast, ZO-1 expression remained consistent across anatomical sites and cycle stages, suggesting that the tight junction scaffold is maintained despite changes in claudin abundance. Together, these results indicate selective, region-specific remodeling of the tight junction’s claudin composition during estrus, without disruption of the ZO-1 scaffold. As this study assessed protein expression and localization rather than barrier function directly, we did not measure epithelial permeability, and any implications for antigen access following intrauterine vaccination remain a hypothesis for future functional studies rather than a conclusion supported by the present data.

## Data Availability

The original contributions presented in the study are included in the article/**Supplementary Material.** Further inquiries can be directed to the corresponding author.

## Supplementary material

Supplementary Figure 1 Chromogenic immunohistochemical validation of tight junction protein localization in the porcine upper reproductive tract. Representative DAB-based immunohistochemistry images demonstrating apical junctional localization of **(A)** Claudin-4, **(B)** Claudin-3, and **(C)** ZO-1 in the distal uterine horn, medial uterine horn, and cervix during estrus and diestrus. Brown DAB signal indicates positive staining; nuclei were counterstained with hematoxylin (blue). Localization patterns were consistent with the confocal immunofluorescence findings presented in **Figures 2**-**4**. Scale bars = 200 µm.

Supplementary Figure 2 Validation of antibody specificity and assessment of background fluorescence. **(A)** Isotype control: Sections were incubated with a mixture of rabbit IgG and mouse IgG κ isotype control antibodies followed by the corresponding secondary antibodies (goat anti-rabbit Alexa Fluor™ 488, green and goat anti-mouse Alexa Fluor™ 568, red). **(B)** Secondary-only control: Sections were incubated with secondary antibodies alone to assess nonspecific binding and tissue autofluorescence. All sections were counterstained with DAPI (blue) and imaged using identical acquisition settings (laser power, gain, and offset) as experimental samples. No specific junctional staining was observed in control conditions. Scale bar = 10 µm.

## References

[1] Krug SM Schulzke JD Fromm M . Tight junction, selective permeability, and related diseases. Semin Cell Dev Biol. (2014) 36:166–76. doi: 10.1016/j.semcdb.2014.09.002 25220018

[2] Pasternak JA Hamonic G Forsberg NM Wheler CL Dyck MK Wilson HL . Intrauterine delivery of subunit vaccines induces a systemic and mucosal immune response in rabbits. Am J Reprod Immunol. (2017) 78:e12732. doi: 10.1111/aji.12732 28771858

[3] Fahey JV Prabhala RH Guyre PM Wira CR . Antigen-presenting cells in the human female reproductive tract: analysis of antigen presentation in pre- and post-menopausal women. Am J Reprod Immunol (New York N Y 1989). (1999) 42:49–57. doi: 10.1111/j.1600-0897.1999.tb00465.x 10429767

[4] Katila T . Post-mating inflammatory responses of the uterus. Reprod Domest Anim. (2012) 47:31–41. doi: 10.1111/j.1439-0531.2012.02120.x 22913558

[5] Rozeboom KJ Troedsson MH Crabo BG . Characterization of uterine leukocyte infiltration in gilts after artificial insemination. J Reprod Fertil. (1998) 114:195–9. doi: 10.1530/jrf.0.1140195 10070347

[6] Kaeoket K Persson E Dalin AM . The influence of pre- and post-ovulatory insemination on sperm distribution in the oviduct, accessory sperm to the zona pellucida, fertilisation rate and embryo development in sows. Anim Reprod Sci. (2002) 71:239–48. doi: 10.1016/s0378-4320(02)00022-2 12047932

[7] Bischof RJ Lee CS Brandon MR Meeusen E . Inflammatory response in the pig uterus induced by seminal plasma. J Reprod Immunol. (1994) 26:131–46. doi: 10.1016/0165-0378(94)90036-1 7932389

[8] Hamonic G Pasternak JA Ng SH Fourie KR Simko OM Deluco B . Assessment of immunological response and impacts on fertility following intrauterine vaccination delivered to swine in an artificial insemination dose. Front Immunol. (2020) 11:1015. doi: 10.3389/fimmu.2020.01015 32536924 PMC7267065

[9] Prior JC . Women’s reproductive system as balanced estradiol and progesterone actions—a revolutionary, paradigm-shifting concept in women’s health. Drug Discov Today: Dis Models. (2020) 32:31–40. doi: 10.1016/j.ddmod.2020.11.005 38826717

[10] Bradley F Stern A Franzén Boger M Mousavian Z Dethlefsen O Kaldhusdal V . Estradiol-mediated enhancement of the human ectocervical epithelial barrier correlates with desmoglein-1 expression in the follicular menstrual phase. Front Endocrinol (Lausanne). (2024) 15:1454006. doi: 10.3389/fendo.2024.1454006 39439565 PMC11493707

[11] Jirik L Joshi S Nazli A Kaushic C . Beyond the barrier: Epithelial cells as immune sentinels in the female genital tract. Am J Reprod Immunol. (2026) 95:e70243. doi: 10.1111/aji.70243 42090241 PMC13148466

[12] Ayehunie S Islam A Cannon C Landry T Pudney J Klausner M . Characterization of a hormone-responsive organotypic human vaginal tissue model: morphologic and immunologic effects. Reprod Sci. (2015) 22:980–90. doi: 10.1177/1933719115570906 25676577 PMC5933095

[13] Ochiel DO Fahey JV Ghosh M Haddad SN Wira CR . Innate immunity in the female reproductive tract: role of sex hormones in regulating uterine epithelial cell protection against pathogens. Curr Womens Health Rev. (2008) 4:102–17. doi: 10.2174/157340408784246395 19644567 PMC2717724

[14] Wira CR Fahey JV Sentman CL Pioli PA Shen L . Innate and adaptive immunity in female genital tract: cellular responses and interactions. Immunol Rev. (2005) 206:306–35. doi: 10.1111/j.0105-2896.2005.00287.x 16048557

[15] Wira CR Grant-Tschudy KS Crane-Godreau MA . Epithelial cells in the female reproductive tract: a central role as sentinels of immune protection. Am J Reprod Immunol. (2005) 53:65–76. doi: 10.1111/j.1600-0897.2004.00248.x 15790340

[16] Capaldo CT Nusrat A . Cytokine regulation of tight junctions. Biochim Biophys Acta. (2009) 1788:864–71. doi: 10.1016/j.bbamem.2008.08.027 18952050 PMC2699410

[17] Fahey JV Wright JA Shen L Smith JM Ghosh M Rossoll RM . Estradiol selectively regulates innate immune function by polarized human uterine epithelial cells in culture. Mucosal Immunol. (2008) 1:317–25. doi: 10.1038/mi.2008.20 19079193 PMC4815904

[18] Denker HW . Back to the future-a 50-year dive into embryo implantation research: cell biological paradox, epithelial cell polarity, and EMT. Biomolecules. (2026) 16:293. doi: 10.3390/biom16020293 41750362 PMC12937988

[19] Classen-Linke I Buck VU Sternberg AK Kohlen M Izmaylova L Leube R . Changes in epithelial cell polarity and adhesion guide human endometrial receptivity: how *in vitro* systems help to untangle mechanistic details. Biomolecules. (2025) 15:1057. doi: 10.3390/biom15081057 40867502 PMC12383966

[20] Capaldo CT Farkas AE Hilgarth RS Krug SM Wolf MF Benedik JK . Proinflammatory cytokine-induced tight junction remodeling through dynamic self-assembly of claudins. Mol Biol Cell. (2014) 25:2710–9. doi: 10.1091/mbc.e14-02-0773 25031428 PMC4161507

[21] Raya-Sandino A Lozada-Soto KM Rajagopal N Garcia-Hernandez V Luissint AC Brazil JC . Claudin-23 reshapes epithelial tight junction architecture to regulate barrier function. Nat Commun. (2023) 14:6214. doi: 10.1038/s41467-023-41999-9 37798277 PMC10556055

[22] Monaco A Ovryn B Axis J Amsler K . The epithelial cell leak pathway. Int J Mol Sci. (2021) 22:7677. doi: 10.3390/ijms22147677 34299297 PMC8305272

[23] González-Mariscal L Betanzos A Ávila-Flores A . MAGUK proteins: structure and role in the tight junction. Semin Cell Dev Biol. (2000) 11:315–24. doi: 10.1006/scdb.2000.0178 10966866

[24] van der Veen RE Bieck M Mezouar N Haucke V Dimke H Lehmann M . Control of renal calcium permeability via a tight junctional claudin switch. Proc Natl Acad Sci USA. (2025) 122:e2512046122. doi: 10.1073/pnas.2512046122 41325524 PMC12704743

[25] Selim MS Narayanan SP Somanath PR . Claudins in retinal disease: beyond barrier integrity to signaling and therapy. Cells. (2026) 15(5):417. doi: 10.3390/cells15050417 41827851 PMC12984813

[26] Gamero-Estevez E Andonian S Jean-Claude B Gupta I Ryan AK . Temporal effects of quercetin on tight junction barrier properties and claudin expression and localization in MDCK II cells. Int J Mol Sci. (2019) 20(19):4889. doi: 10.3390/ijms20194889 31581662 PMC6801663

[27] González-Mariscal L Betanzos A Nava P Jaramillo BE . Tight junction proteins. Prog Biophys Mol Biol. (2003) 81:1–44. doi: 10.1016/s0079-6107(02)00037-8 12475568

[28] Hamonic G Pasternak JA Forsberg NM Käser T Wilson HL . Expression of pattern recognition receptors in porcine uterine epithelial cells *in vivo* and in culture. Vet Immunol Immunopathol. (2018) 202:1–10. doi: 10.1016/j.vetimm.2018.06.006 30078581

[29] Van Itallie CM Fanning AS Bridges A Anderson JM . ZO-1 stabilizes the tight junction solute barrier through coupling to the perijunctional cytoskeleton. Mol Biol Cell. (2009) 20:3930–40. doi: 10.1091/mbc.e09-04-0320 19605556 PMC2735491

[30] Braniste V Leveque M Buisson-Brenac C Bueno L Fioramonti J Houdeau E . Oestradiol decreases colonic permeability through oestrogen receptor beta-mediated up-regulation of occludin and junctional adhesion molecule-A in epithelial cells. J Physiol. (2009) 587:3317–28. doi: 10.1113/jphysiol.2009.169300 19433574 PMC2727039

[31] Burek M Steinberg K Förster CY . Mechanisms of transcriptional activation of the mouse claudin-5 promoter by estrogen receptor alpha and beta. Mol Cell Endocrinol. (2014) 392:144–51. doi: 10.1016/j.mce.2014.05.003 24846172

[32] Xia M Dong S Cao J Wang J Wang C . Identification and validation of a novel estrogen-related model for breast cancer to predict the prognosis. Transl Cancer Res. (2026) 15:107. doi: 10.21037/tcr-2025-1409 41815145 PMC12971564

[33] Kozieł MJ Kowalska K Piastowska-Ciesielska AW . Claudins: new players in human fertility and reproductive system cancers. Cancers. (2020) 12:711. doi: 10.3390/cancers12030711 32197343 PMC7140004

[34] Pasternak JA Kent-Dennis C Van Kessel AG Wilson HL . Claudin-4 undergoes age-dependent change in cellular localization on pig jejunal villous epithelial cells, independent of bacterial colonization. Mediators Inflammation. (2015) 2015:263629. doi: 10.1155/2015/263629 25948883 PMC4407623

[35] Bauersachs S Wolf E . Immune aspects of embryo-maternal cross-talk in the bovine uterus. J Reprod Immunol. (2013) 97:20–6. doi: 10.1016/j.jri.2012.11.002 23432868

[36] Mendoza-Rodríguez CA González-Mariscal L Cerbón M . Changes in the distribution of ZO-1, occludin, and claudins in the rat uterine epithelium during the estrous cycle. Cell Tissue Res. (2005) 319:315–30. doi: 10.1007/s00441-004-1010-7 15558325

[37] Pan XY Wang B Che YC Weng ZP Dai HY Peng W . Expression of claudin-3 and claudin-4 in normal, hyperplastic, and Malignant endometrial tissue. Int J Gynecol Cancer. (2007) 17:233–41. doi: 10.1111/j.1525-1438.2006.00748.x 17291259

[38] Pasternak JA Hamonic G Van Kessel J Wheler CL Dyck MK Wilson HL . Intrauterine vaccination induces a dose-sensitive primary humoral response with limited evidence of recall potential. Am J Reprod Immunol. (2018) 80:e12855. doi: 10.1111/aji.12855 29607560

[39] Choudhary P Fourie KR Ng S Hamonic G Bérubé N Popowych Y . Intrauterine immunizations trigger antigen-specific mucosal and systemic immunity in pigs and passive protection in suckling piglets. Vaccine. (2021) 39(42):6322–32. doi: 10.1016/j.vaccine.2021.08.080 34535320

[40] Steinman RM Inaba K Turley S Pierre P Mellman I . Antigen capture, processing, and presentation by dendritic cells: recent cell biological studies. Hum Immunol. (1999) 60:562–7. doi: 10.1016/s0198-8859(99)00030-0 10426272

[41] Bulmer JN Williams PJ Lash GE . Immune cells in the placental bed. Int J Dev Biol. (2010) 54:281–94. doi: 10.1387/ijdb.082763jb 19876837

[42] Kaeoket K Persson E Dalin AM . Corrigendum to "The sow endometrium at different stages of the oestrus cycle: studies on morphological changes and infiltration by cells of the immune system." [Anim. Reprod. Sci. 65 (2001) 95-114. Anim Reprod Sci. (2002) 73:89–107. doi: 10.1016/s0378-4320(02)00126-4 12220821

